# Pembrolizumab-Induced Isolated Adrenocorticotropic Hormone (ACTH) Deficiency 

**DOI:** 10.7759/cureus.52235

**Published:** 2024-01-13

**Authors:** Khalid Alfares, Hye Jeong Han

**Affiliations:** 1 Endocrinology, Diabetes and Metabolism, King Abdulaziz University Faculty of Medicine, Jeddah, SAU; 2 Endocrinology, Diabetes and Metabolism, Henry Ford Health System, Detroit, USA; 3 Internal Medicine, Henry Ford Health System, Detroit, USA

**Keywords:** pituitary hormones, chemotherapy-related toxicity, low cortisol, pd-1 inhibitors, pembrolizumab, adrenal insufficiency, acth deficiency

## Abstract

Pembrolizumab is a programmed death 1 receptor (PD-1) inhibitor. It is used as immunotherapy in various cancers, including metastatic melanoma, non-small cell lung cancer, and, notably, high-risk triple-negative breast cancer. We discuss a case of a 44-year-old female with a past medical history of triple-negative breast cancer who presented with a chief complaint of poor oral intake and fatigue after her fourth cycle of pembrolizumab therapy. The patient was diagnosed with pembrolizumab-induced isolated secondary adrenal insufficiency (AI) and was treated with corticosteroids with improvement in her symptoms. Secondary AI due to pembrolizumab use is a rare yet potentially life-threatening complication. If initial serum cortisol is borderline low, as observed in our patient, repeated testing within shorter intervals should be considered to optimize patient outcomes.

## Introduction

Pembrolizumab is a programmed death 1 receptor (PD-1) inhibitor and is used as immunotherapy in different cancers, including metastatic melanoma, non-small cell lung cancer, and, notably, high-risk triple-negative breast cancer [1-3]. In a study by Ariyasu et al. involving a total of 168 patients who received anti-PD-1 therapy alone or in combination with anti-CTLA-4, only five patients developed secondary adrenal insufficiency (AI), and only one of those five patients had a pituitary abnormality on MRI [4]. We only found a few case reports of pembrolizumab-induced isolated adrenocorticotropic hormone (ACTH) deficiency in the literature. One of them involved a metastatic colon cancer patient who developed secondary AI after two cycles of pembrolizumab [5]. Late-onset secondary AI can occur even after 15 months of therapy, as reported in a patient with stage IV large-cell lung carcinoma who developed secondary AI after completing 24 cycles of pembrolizumab [6]. In this report, we present a rare case of pembrolizumab-induced isolated ACTH insufficiency.

## Case presentation

A 44-year-old female with a past medical history of cerebral palsy and triple-negative breast cancer initially presented to the outpatient oncology office for routine evaluation. She had recently completed chemotherapy and was started on immunotherapy with pembrolizumab. Her oncologist was monitoring her periodically for immune-related adverse events (irAE) associated with pembrolizumab therapy. After the second and third cycles of pembrolizumab, random cortisol levels were 8.8 mcg/dL and 5.8 mcg/dL, respectively. Following her fourth cycle, she started complaining of poor oral intake, generalized fatigue, and difficulty ambulating. On the subsequent visit, labs revealed an insidious drop of random cortisol level to 0.4 mcg/dL. She was advised to go to the emergency department. Upon presentation, the patient was noted to be confused but hemodynamically stable. Serum morning cortisol was rechecked and found to be 0.4 mcg/dL, confirming AI. The standard dose cosyntropin stimulation test (250 mcg) revealed an unsatisfactory response, with serum cortisol levels of 6.4 mcg/dL at 30 and 8.5 mcg/dL at 60 minutes, respectively. Plasma ACTH was 2 pg/ml (normal range: 7-63 pg/mL) consistent with secondary AI. Dehydroepiandrosterone-sulfate (DHEAS), an ACTH-dependent adrenal androgen, was low at 8.1 mcg/dL. Serum estradiol was less than 20 pg/ml due to known primary ovarian failure from chemotherapy. Her gonadotrophin levels were appropriately elevated at the post-menopausal range: LH was 34.8 mIU/ml, and FSH was 56.2 mIU/ml. Thyroid-stimulating hormone (TSH) and free thyroxine (T4) were within normal ranges at 1.38 uIU/mL and 0.73 ng/dL, respectively (Table 1).

**Table 1:** Lab results ACTH: adrenocorticotropic hormone; DHEAS: dehydroepiandrosterone-sulfate; LH: luteinizing hormone; FSH: follicle-stimulating hormone; TSH: thyroid-stimulating hormone

Labs	Normal range	Patient values
AM cortisol	4.3-22.4 mcg/dL	<0.4 mcg/dL
Cortisol post cosyntropin stimulation test (30 minutes)	≥18 mcg/dL	6.4 mcg/dL
Cortisol post cosyntropin stimulation test (60 minutes)	≥18 mcg/dL	8.5 mcg/dL
Plasma ACTH	7-63 pg/ml	2 pg/ml
DHEAS	27-240 mcg/dL (age- and gender-specific)	8.1 mcg/dL
Serum estradiol	<48 pg/ml (post-menopausal range)	<20 pg/mL
LH	10.9-58.6 mIU/ml (post-menopausal range)	34.8 mIU/ml
FSH	16.7-113.6 mIU/ml (post-menopausal range)	56.2 mIU/ml
TSH	0.45-5.33 uIU/ml	1.38 uIU/mL
Free T4	0.61-1.44 ng/dL	0.73 ng/dL

Pituitary/sellar MRI did not reveal any abnormality or lesion. Her workup confirmed isolated ACTH deficiency. The patient was administered a stress-dose glucocorticoid replacement for three days. On day four of admission, she was transitioned to a physiological maintenance dose of hydrocortisone 15 mg in the morning and 5 mg in the afternoon, with immediate improvement in her appetite, mentation, and energy levels.

## Discussion

**Figure 1:**
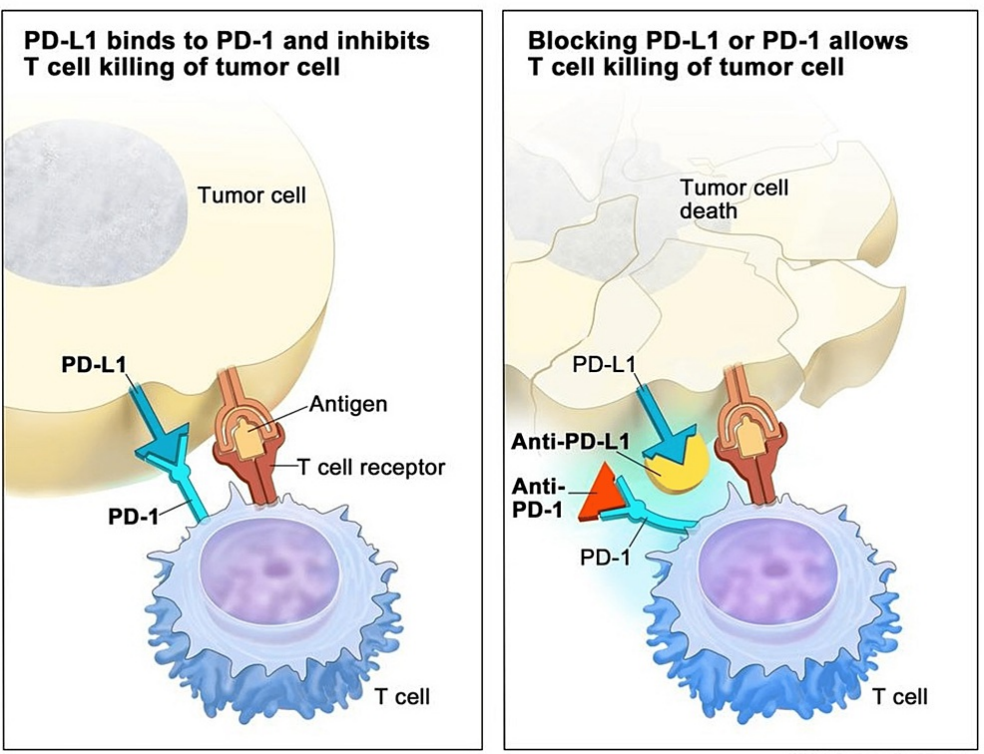
PD-1 inhibitors and PD-L1 inhibitors - mechanisms of action The binding of PD-L1 to PD-1 keeps T cells from killing tumor cells in the body (left panel). Blocking the binding of PD-L1 to PD-1 with an immune checkpoint inhibitor (anti-PD-L1 or anti-PD-1) allows the T cells to kill tumor cells (right panel) © 2023 Terese Winslow LLC. Permission was obtained from the original authors to use this image PD-1: programmed cell death protein 1; PD-L1: programmed death-ligand 1

This case demonstrates the importance of maintaining a high index of suspicion to screen for AI in patients receiving immune checkpoint inhibitor (ICI) therapy. Pembrolizumab is a PD-1 that plays a vital role in inhibiting immune responses and promoting self-tolerance by modulating the activity of T-cells, activating apoptosis of antigen-specific T cells, and inhibiting apoptosis of regulatory T cells [7]. It is used as immunotherapy in various cancers, including metastatic melanoma, non-small cell lung cancer, and, notably, high-risk triple-negative breast cancer, as in our patient (Figure 1).

One under-recognized side effect of pembrolizumab is isolated secondary AI. Secondary AI due to ACTH deficiency appears in 0.8% of patients treated with ICI, mainly in males in the seventh decade of life and approximately seven months after starting immunotherapy. Its pathogenesis is unknown in most patients, although it has also been suggested that it could be caused by autoimmunity to corticotrophs (Table 2) [8].

**Table 2:** Clinical characteristics of the different forms of pituitary involvement in cancer patients treated with ICI antibodies* *[9] Anti-CTLA-4: anti-cytotoxic T-lymphocyte antigen 4; Anti-PD-1: anti-programmed cell death protein 1; ICI: immune checkpoint inhibitor; MRI: magnetic resonance imaging

	Hypophysitis	Isolated ACTH deficiency (IAD)
Type of ICI	Anti-CTLA-4 (ipilimumab)	Anti-PD-1 (nivolumab)
Prevalence	10-17%	<1%
Time since ICI therapy initiation	9 weeks	7 months
Main symptom	Headache	Fatigue
Hyponatremia	47%	24-68%
Pituitary hormone alterations	>2	ACTH
Pituitary MRI	Pituitary enlargement	Normal

To the best of our knowledge, there is currently no protocol on the frequency of monitoring serum cortisol in patients on pembrolizumab therapy. The median onset of developing primary AI is 10 weeks. However, the onset of developing secondary AI is not well established. This unique case of pembrolizumab-induced secondary AI highlights the importance of having a low threshold to screen for AI when a patient on pembrolizumab presents with symptoms of poor oral intake and generalized weakness. If the initial serum cortisol is borderline low, as noted in our patient, repeated testing within shorter intervals should be considered as an insidious drop in cortisol levels can occur. Failure to diagnose and treat AI can result in an adrenal crisis, which carries a high risk of mortality. If an adrenal crisis is suspected, prompt treatment should be initiated, and the patient should be started on stress-dose glucocorticoid replacement, followed by maintenance therapy when clinically stable.

## Conclusions

Pembrolizumab has demonstrated considerable effectiveness in treating various types of cancer. However, its use is associated with specific endocrine-related side effects, particularly secondary AI, which is a rare yet potentially life-threatening complication. If initial serum cortisol is borderline low, as noted in our patient, repeated testing within shorter intervals should be considered to optimize patient outcomes.
